# Efficacy of Abatacept for Arthritis in Patients with an Overlap Syndrome between Rheumatoid Arthritis and Systemic Lupus Erythematosus

**DOI:** 10.1155/2013/697525

**Published:** 2013-11-14

**Authors:** Kei Ikeda, Yoshie Sanayama, Sohei Makita, Junichi Hosokawa, Mieko Yamagata, Daiki Nakagomi, Katsuhiko Takabayashi, Hiroshi Nakajima

**Affiliations:** Department of Allergy and Clinical Immunology, Chiba University Hospital, 1-8-1 Inohana, Chuo-ku, Chiba-shi, Chiba 260-8670, Japan

## Abstract

*Introduction.* This study aimed to investigate the efficacy of abatacept for arthritis in patients with rhupus, an overlap syndrome between rheumatoid arthritis (RA) and systemic lupus erythematosus (SLE). *Methods.* Patients who fulfilled both the 2010 ACR/EULAR criteria for RA classification and the 1997 ACR revised criteria for classification of SLE and received abatacept treatment for arthritis were retrospectively studied. *Results.* Six rhupus patients who fulfilled the inclusion criteria above were identified. All patients had active arthritis despite receiving antirheumatic drugs including methotrexate when abatacept was initiated. Clinical Disease Activity Index (CDAI) significantly decreased between baseline and 12 weeks (*P* = 0.028) and remained low through 24 weeks. All patients achieved either a good or moderate response according to the EULAR response criteria at 24 weeks. Health Assessment Questionnaire-Disability Index (HAQ-DI) also significantly decreased between baseline and 24 weeks (*P* = 0.043). In addition, the levels of immunoglobulin G and anti-DNA antibody significantly decreased between baseline and 24 weeks (*P* = 0.028 and *P* = 0.043, resp.). *Conclusions.* Treatment with abatacept is likely to be efficacious in patients with rhupus whose arthritis is refractory to methotrexate. In addition, abatacept may have a moderate effect on abnormal antibody production in rhupus patients.

## 1. Introduction

The clinical coexistence of rheumatoid arthritis (RA) and systemic lupus erythematosus (SLE) is a rare occurrence frequently referred to as “rhupus syndrome” [1]. Increasing evidence suggests that arthritis in patients with rhupus can cause joint damage indistinguishable from that of RA, requiring aggressive treatment [2–5]. However, TNF antagonists, which are the most potent agents in preventing joint damage in RA when used in combination with methotrexate (MTX), can induce production of autoantibodies characteristic to SLE such as antinuclear antibodies (ANA) or anti-DNA antibodies [6, 7]. Less frequently but more importantly, TNF antagonists can cause lupus manifestations in RA [6–10] and rhupus syndrome [11].

Abatacept is a fully human, soluble fusion protein that consists of the extracellular domain of human cytotoxic T-lymphocyte antigen 4 (CTLA-4) and the Fc portion of IgG1, which selectively modulates the CD80/CD86:CD28 costimulatory signals and interactions between activated T cells and antigen presenting cells (APCs). The use of abatacept in patients with RA is associated with sustained efficacy both in disease activity and in radiographic progression without inducing autoantibody production [12–16]. Abatacept treatment has been explored for its efficacy in other T cell-mediated diseases such as ankylosing spondylitis [17, 18]. Moreover, a recent phase IIb randomized, double-blind, placebo-controlled trial showed modest but significant efficacy of abatacept against polyarthritis in patients with non-life-threatening SLE [19]. However, abatacept treatment in rhupus patients has not been reported.

In this study, we retrospectively assessed the efficacy of abatacept in six rhupus patients with active arthritis but not with life-threatening lupus manifestations.

## 2. Materials and Methods

### 2.1. Patients

Medical records in the Department of Allergy and Clinical Immunology, Chiba University Hospital were thoroughly reviewed to identify patients who received abatacept treatment for arthritis and also fulfilled both the 2010 ACR/EULAR criteria for RA classification and the 1997 ACR revised criteria for classification of SLE. In order to ensure the inclusion of patients with genuine overlap, patients were excluded when the arthritis was better explained by SLE than by RA, and arthritis was not counted when SLE was classified. All patients gave a written consent for their clinical information to be published and the study procedures were approved by the Ethics Committee of Chiba University.

### 2.2. Statistical Analysis

Statistical analysis was performed using SPSS version 16.0J (IBM Japan, Tokyo, Japan). As all data were not normally distributed, data were summarized with medians and were analyzed using nonparametric tests (Wilcoxon's signed-rank test). *P* values less than 0.05 were considered significant.

## 3. Results

### 3.1. Demographics and Disease Characteristics of RA

**Figure 1:**
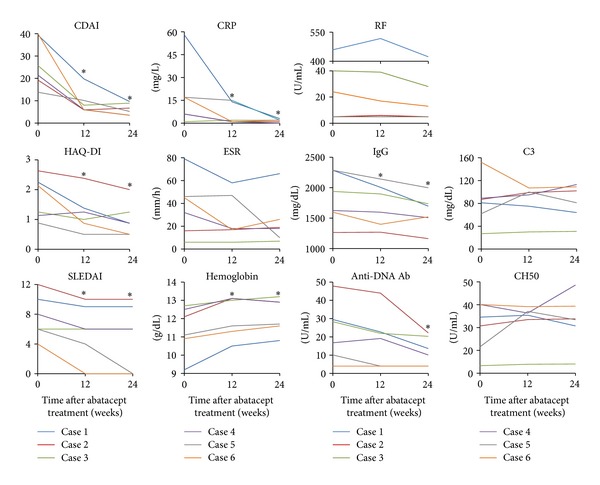
Changes in clinical indices and laboratory tests reflecting disease activity of RA and/or SLE in each case during 24 weeks after abatacept treatment. *P < 0.05, Wilcoxon's signed-rank test. Comparisons were made against baseline values. RA: rheumatoid arthritis; SLE: systemic lupus erythematosus; CDAI: Clinical Disease Activity Index; HAQ-DI: Health Assessment Questionnaire-Disability Index; SLEDAI: systemic lupus erythematosus disease activity index; CRP: C-reactive protein; ESR: erythrocyte sedimentation rate; RF, rheumatoid factor; IgG: immunoglobulin G; Anti-DNA Ab: anti-DNA antibody.

Six patients who fulfilled the above mentioned inclusion criteria were identified. Demographics and disease characteristics of RA before abatacept administration of these patients are summarized in Table 1. All patients were Japanese females with a median age of 57.5 years. Four patients had an onset of arthritis symptoms which preceded the diagnosis of SLE. Three patients were seronegative (i.e., both rheumatoid factor [RF] and anticitrullinated protein antibody [ACPA] were negative) at baseline although one of them (Case 5) was positive for RF at the time of the diagnosis of SLE. Five patients had at least one erosive lesion on radiograph that was typical of RA. Median level of C-reactive protein (CRP) at baseline was relatively low (11.5 mg/L) as compared to median Clinical Disease Activity Index (CDAI) (23.55) (Figure 1). All patients underwent musculoskeletal ultrasonography for the assessment of synovial inflammation before abatacept treatment. All patients had increased PD signals within intra-articular synovium (i.e., active intra-articular synovitis) in at least one joint region, and five out of six patients had increased PD signals within tenosynovium as well (i.e., active tenosynovitis) in at least one joint region. 

### 3.2. Disease Characteristics of SLE

Disease characteristics of SLE before abatacept treatment are summarized in Table 2. No patient had previously experienced severe organ manifestations of SLE such as nephritis or neuropsychiatric lupus, and arthritis was the major disease manifestation when abatacept was introduced. Sjogren's syndrome was the most common concomitant autoimmune disease other than RA or SLE (*n* = 4), followed by chronic thyroiditis (*n* = 2) and scleroderma (*n* = 1). All patients were positive for antinuclear antibody and five patients were positive for anti-DNA antibody at the baseline. Antidouble stranded DNA antibody in Case 6 was positive when the patient was diagnosed with SLE but turned negative under treatment. Other autoantibodies, which, were positive at baseline were anti-Ro antibody (*n* = 4), anti-U1-RNP antibody (*n* = 3), anti-La antibody (*n* = 2), anti-thyroid peroxidase antibody (*n* = 2), anti-cardiolipin antibody (*n* = 1), and lupus anticoagulant (*n* = 1). Reflecting the lack of severe organ manifestations, clinically significant hypocomplementemia was only present in one patient (Case 3).

### 3.3. Previous Treatment

Five patients were receiving a small dose of prednisolone (median 4.5 mg/day), whereas one patient (Case 1) was not because the patient did not agree to receive corticosteroid therapy. All patients were receiving treatment with MTX although half of them (*n* = 3) discontinued MTX due to either cytopenia, elevation of liver enzymes, or lack of efficacy when abatacept therapy was initiated. Three patients were receiving a calcineurin inhibitor such as tacrolimus (*n* = 2) or cyclosporine A (*n* = 1), effectiveness of which was insufficient. Two patients had previously received a TNF antagonist, which was not effective in either case. One patient (Case 1) received adalimumab before the diagnosis of SLE. Although the patient had already tested positive for anti-nuclear antibody (ANA) and anti-DNA antibody before receiving adalimumab and it was discontinued after only four injections 7 months before the development of her major lupus symptoms; the TNF*α* blockade could have been the trigger for the onset of SLE in her case. No patient had received rituximab because the use of rituximab has been approved for neither RA nor SLE in Japan.

### 3.4. Efficacy of Abatacept for Arthritis

As shown in Figure 1, CDAI and CRP significantly decreased after 12 weeks of abatacept treatment (median CDAI 23.55 versus 7 and *P* = 0.028; median CRP 11.5 mg/L versus 1.5 mg/L and *P* = 0.046) and remained low through 24 weeks (median CDAI 5.95; median CRP 2.0 mg/L). Four patients achieved a good or moderate response according to EULAR response criteria at 12 weeks (three good and one moderate) and all patients achieved a good or moderate response at 24 weeks (two good and four moderate). Health Assessment Questionnaire-Disability Index (HAQ-DI) also significantly decreased from median of 2.13 at baseline to median of 0.87 at 12 weeks (*P* = 0.046) and median of 0.5 at 24 weeks (*P* = 0.043). In addition, matrix metalloproteinase 3 (MMP-3) also significantly decreased from median of 132 ng/mL at baseline to median of 88 ng/mL at 12 weeks (*P* = 0.028) and median of 76.7 ng/mL at 24 weeks (*P* = 0.028) (data not shown). Prednisolone dose was decreased slightly but successfully by 1 mg/day in Case 3 and 6 before 24 weeks. These results suggest that abatacept is efficacious in the treatment of arthritis for patients with rhupus.

### 3.5. Efficacy of Abatacept on Nonarticular Lupus Manifestations

As shown in Figure 1, Systemic Lupus Erythematosus Disease Activity Index (SLEDAI) significantly decreased from median of 7 at baseline to median of 6 at 12 and 24 weeks (*P* = 0.039 and 0.042, resp.), mostly reflecting the remission induction of arthritis in Cases 5 and 6. Other major symptoms which, upon improvement, decreased SLEDAI were fever in Case 1 and rash in Case 5. Among laboratory tests for lupus activity, hemoglobin significantly increased from median of 11.6 g/dL to median of 12.3 g/dL at 12 and 24 weeks (*P* = 0.028). None of the six patients had thrombocytopenia and only one had leukocytopenia at baseline, which did not improve with abatacept treatment. The serum levels of immunoglobulin G (IgG) and anti-DNA antibody also significantly decreased from baseline to 24 weeks (IgG, median 1,782 mg/dL to 1,609.5 mg/dL, and *P* = 0.028; anti-DNA antibody, median 22.55 U/mL to 11.85 U/mL, and *P* = 0.043). However, changes in C3 levels and CH50 were variable and were not statistically significant. These data suggest that the efficacy of abatacept on nonarticular lupus manifestation at low disease activity states may be absent but abatacept may have moderate effect on abnormal antibody production in rhupus patients.

### 3.6. Adverse Events

One patient (Case 5) experienced mild olecranon bursitis in the elbow after 2nd administration of abatacept. The bursitis was considered to be self-limiting or infectious, since swelling in the other joints was improving and the symptom subsided after 1-week treatment with oral antibiotics. The 3rd administration was postponed for a week, but abatacept was restarted without relapse. No other adverse events, including exacerbation of any lupus manifestations or concomitant autoimmune conditions, have been reported for a median follow-up period of 15 months (range 7–22).

### 3.7. Case Report (Case 3)

A 60-year-old woman was admitted to the Department of Allergy and Clinical Immunology, Chiba University Hospital, on 13th of November, 2009, for the treatment of RA and SLE. She had been diagnosed with RA since 1996, when she had arthritis in hands and tested positive for RF, but had only received Chinese herbal medicine since the diagnosis. She was diagnosed with duodenal cancer and SLE in August, 2009, when she was admitted to another hospital for the investigation of intermittent fever, pancytopenia, multiple lymphadenopathy, and congestive heart failure. After receiving treatment for anemia and congestive heart failure with blood transfusion and diuretics, the patient underwent distal gastrectomy and proximal duodenectomy, on 28th of October, 2009 at the Department of Frontier Surgery, Chiba University Hospital, without any complication. The histopathological diagnosis was papillary adenocarcinoma. 

**Figure 2:**
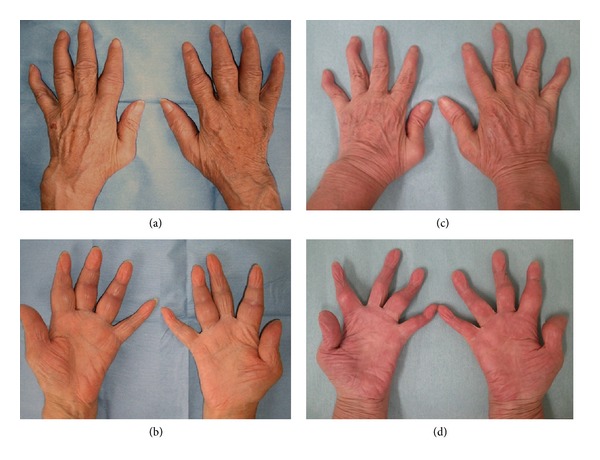
Joint swelling of hands in Case 3 before and after treatment. Dorsal aspects (a, c) and palmer aspects (b, d) of the hand are shown. Swelling in fingers and wrists before treatment (a, b) markedly improved after 24 weeks of abatacept treatment (c, d), leaving swan-neck deformity.

On admission to our department, the patient had a low grade fever, lymphadenopathy in the neck and bilateral inguinal areas, and markedly swollen but only slightly tender fingers and wrists (Figures 2(a) and 2(b)). Blood tests revealed bicytopenia (white blood cell count 1,900/mm^3^, hemoglobin 10.2 g/dL, platelet count 405 × 10^3^/mm^3^), acute inflammatory response (CRP 32 mg/L, erythrocyte sedimentation rate [ESR] 48 mm/h), hypergammaglobulinemia (IgG 2,395 mg/dL), abnormal coagulation (prothrombin time-international normalized ratio [PT-INR] 1.14, activated partial thromboplastin time [APTT] 40.1 sec, D-Dimer 23.2 *μ*g/mL), decreased levels of complements (C3 29 mg/dL, C4 5 mg/dL, CH_50_ 7 U/mL), and the presence of autoantibodies (ANA x320 speckled pattern, IgG anti-DNA antibody 20.3 U/mL, *β*2-glycoprotein 1-dependent IgG anti-cardiolipin antibody 16 U/mL, RF 188 U/mL, and ACPA >100 U/mL). Antibodies to extractable nuclear antigens such as Sm, U1-RNP, Ro/SS-A, and La/SS-B were all negative. 

**Figure 3:**
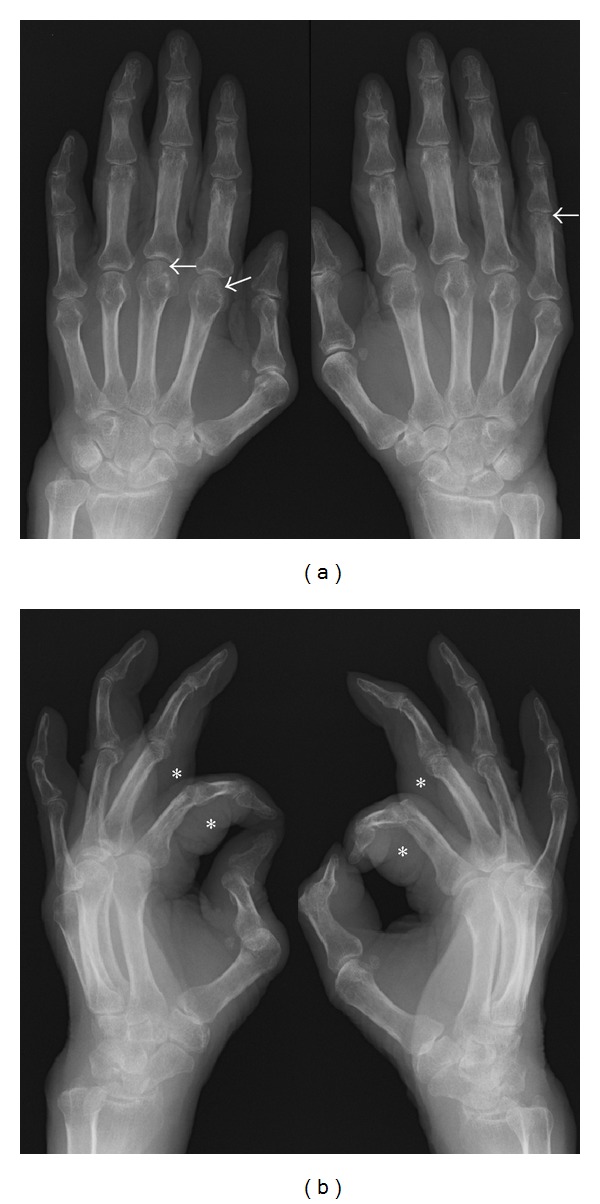
Plain radiographs of hands in Case 3. Plain radiographs of hands before treatment in the anterior-posterior view (a) and the oblique view (b). Arrows indicate small erosions in the heads of left 2nd and 3rd metacarpal bones and right proximal phalanx. Markedly thickened soft tissues are demonstrated (asterisks).

**Figure 4:**
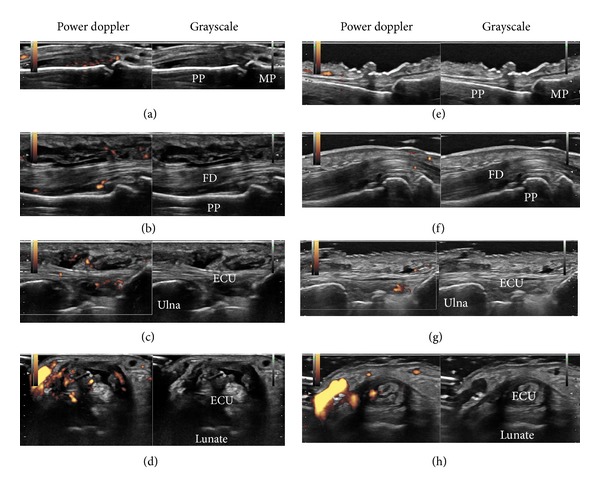
Ultrasound images of hands in Case 3 before and after treatment. Shown are the representative ultrasound images of the left hand before treatment (a–d) and after 24 weeks of abatacept treatment (e–h). Ultrasound images before treatment reveal moderate synovial hypertrophy with moderate Doppler signals in the 3rd proximal interphalangeal joint (a) and severe synovial hypertrophy with moderate Doppler signals in the flexor tendon of the 3rd finger (b) and extensor carpi ulnaris longitudinal view (c) (see also webvideo 1) and transverse view (d) (see also webvideo 2). Corresponding ultrasound images after abatacept treatment demonstrate marked improvement (e–h). PP: proximal phalanx; MP: middle phalanx; FD: flexor digitorum; ECU: extensor carpi ulnaris.

Hand radiographs showed soft tissue swelling, joint space narrowing, and bone erosions (Figure 3). Musculoskeletal ultrasound of the left hand revealed both intra-articular and tenosynovitis with increased PD signals (Figures 4(a)–4(d), [see also webvideo 1, 2] see Supplementary Material available online at http://dx.doi.org/10.1155/2013/697525). Echocardiogram and computed tomography (CT) scan of chest showed a small amount of pericardial effusion. CT scan of abdomen and magnetic resonance imaging of brain were unremarkable.

**Figure 5:**
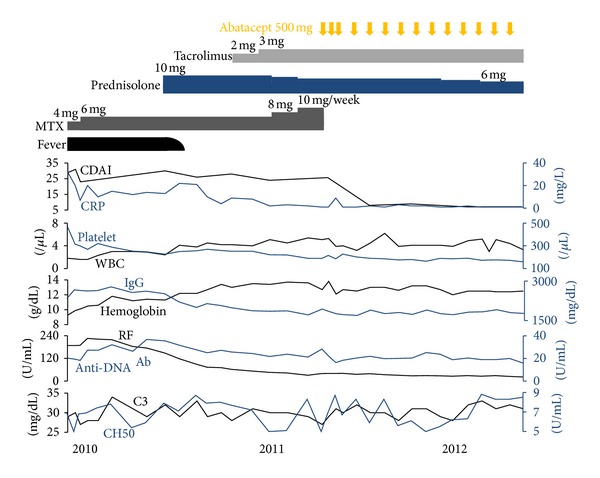
Treatment summary and clinical course of Case 3. MTX: methotrexate; CDAI: Clinical Disease Activity Index; CRP: C-reactive protein; WBC: white blood cell count; IgG: immunoglobulin G; RF: rheumatoid factor; Anti-DNA Ab: anti-DNA antibody; C3: complement component 3; CH50: 50% hemolytic complement activity of serum.1. Panush et al. [1].

The patient fulfilled both the 2010 ACR/EULAR Criteria for the Classification of RA (arthritis in more than ten joints, elevated levels of ESR and CRP, highly positive RF and ACPA, and disease duration of longer than 6 months) and the 1997 ACR Revised Classification Criteria of SLE (Pericarditis, leukocytopenia/lymphocytopenia, anti-DNA antibody/anti-phospholipid antibody, and anti-nuclear antibody). Because she did not have severe organ involvement due to SLE, the treatment for RA was initiated with a small dose of MTX (4 mg/week) given the presence of bicytopenia. CDAI gradually decreased from 35 to 23 with an increase to 6 mg/week of MTX and she was discharged although low grade fever, bicytopenia, and serological activity of lupus persisted (Figure 5). 

A small dose of prednisolone (10 mg/day) was added to the treatment in July 2010 because of a gradual worsening of arthritis, persistent fever, and increased levels of anti-DNA antibody. Although the lupus-like manifestations, including fever, bicytopenia, and increased levels of anti-DNA antibody, improved after corticosteroid therapy; severe joint swelling persisted even after tacrolimus was added to the regimen and MTX was increased to 10 mg/week.

Due to insufficient effectiveness, along with concerns regarding elevated liver enzymes, MTX was replaced by abatacept, in April 2011. Joint swelling markedly improved (Figures 2(c) and 2(d)) and the patient achieved CDAI/SDAI/DAS28 remission after 12 weeks of abatacept treatment (Figure 1, Case 3). Intra-articular and tenosynovitis on ultrasound also improved markedly during the 24 weeks of abatacept treatment (Figures 4(e)–4(h)). Corticosteroid dose was reduced successfully to 6 mg/day in February 2012 and the patient has remained in remission for 14 months.

## 4. Discussion

This is the first report of rhupus patients treated with abatacept for arthritis. All rhupus patients whose arthritis was refractory to MTX and other antirheumatic agents such as TNF antagonists and calcineurin inhibitors achieved a moderate or better response after receiving abatacept treatment. Although this result cannot be directly compared with data from clinical trials, the efficacy of abatacept for arthritis in rhupus patients seems to be at least as good as that in MTX-resistant and biologics-naive RA patients [12, 15]. Considering the potentially detrimental effect of TNF antagonists on lupus manifestation, our data support the preferential choice of abatacept in a patient with rhupus syndrome whose arthritis is refractory to MTX. Whether this also applies to RA patients with positive ANA or anti-DNA antibody without clinical lupus manifestation is a matter of interest, and a comparison between different agents in this subpopulation is needed to address this question.

The relatively safe profile of abatacept as compared to other biological agents for infection in RA patients has been shown in clinical trials and a meta-analysis [12, 20]. Although the bursitis which Case 5 developed in this study could have been due to infection, this event required neither hospitalization nor intravenous administration of antibiotics and did not recur after readministration of abatacept. We think this nonserious adverse event, which could have been infectious, does not necessarily raise a concern about the relative safety of abatacept as compared to other biological agents, but further investigation is nonetheless needed for rhupus patients.

Ultrasound revealed active intra-articular synovitis in all cases and active tenosynovitis in the majority of our cases. The latter lesions may represent the pathology characteristic to SLE rather than RA according to the previous studies which showed high prevalence of tenosynovitis in lupus patients [4, 5, 21]. Although the absence of tenosynovitis did not influence the efficacy of abatacept in our small number of rhupus patients, the discrimination between intra-capsular- and extra-capsular-dominant patients using ultrasound may be informative in the prediction of effectiveness of antirheumatic and immunosuppressive agents for arthritis in RA and SLE. A large-scale prospective study, however, would be necessary to prove this hypothesis.

In contrast to the efficacy of abatacept on arthritis, its efficacy on non-articular lupus manifestations was marginal in our case series, which is consistent with the previous study in patients with non-life-threatening SLE [19]. However, the statistically significant decrease in the levels of IgG and anti-DNA antibody in our cases may reflect the effect of abatacept on autoantibody production in SLE. Because T cell-APC interaction is an attractive target in the pathogenesis of SLE [22–25], more severe cases may respond to abatacept treatment. Accumulation of such cases may justify future trials to identify the subset of SLE patients who may benefit from abatacept treatment. 

## 5. Conclusions

Treatment with abatacept is likely to be efficacious in patients with rhupus whose arthritis is refractory to methotrexate. In addition, abatacept may have a moderate effect on abnormal antibody production in rhupus patients.

## Supplementary Material

Ultrasound images of extensor carpi ulnaris in the right hand before abatacept treatment show severe tenosynovial hypertrophy accompanied by moderate Doppler signals both in the longitudinal view (webvideo 1) and in the transverse view (webvideo 2).

## Figures and Tables

**Table 1:** Patient demographics and disease characteristics of RA before abatacept treatment.

Case number	Sex	Age (year)	Duration of arthritis (month)	RF (U/mL)	ACPA (U/mL)	Erosion on X-ray typical for RA	TJC (/28)	SJC (/28)	Patient global VAS (mm)	Physician global VAS (mm)	DAS28 -ESR	MMP-3 (ng/mL)	Intra-articular synovitis with PD signals	Teno-synovitis with PD signals	Predni-solone (mg/day)	DMARD/ immunosuppressant in combination	Previous DMARD/ immunosuppressant
1	F	29	38	459	110	(+)	16	10	60	73	7.02	116	(+)	(+)	0	MTX	Adalimumab
2	F	66	85	<5	<0.6	(+)	0	5	77	65	3.64	175	(+)	(−)	7	Tacrolimus	MTX
3	F	60	183	40	>100	(+)	5	11	48	49	4.11	87.6	(+)	(+)	8	Tacrolimus	MTX
4	F	58	163	<5	<0.6	(+)	0	12	51	43	4.11	106	(+)	(+)	5	MTX	None
5	F	53	23	<5	0.7	(+)	0	2	25	40	3.08	148	(+)	(+)	4	MTX	Etanercept
6	F	57	108	25	>100	(−)	9	17	66	73	6.42	160	(+)	(−)	2	Cyclosporin A	MTX

RA: rheumatoid arthritis; RF: rheumatoid factor; ACPA: anticitrullinated peptide antibody; TJC: tender joint count; SJC: swollen joint count; VAS: visual analogue scale; DAS: Disease Activity Score; ESR: erythrocyte sedimentation rate; MMP: matrix metalloproteinase; PD: power Doppler; SLE: systemic lupus erythematosus; DMARD: disease-modifying antirheumatic drug; F: female; MTX: methotrexate.

**Table 2:** Disease characteristics of SLE before abatacept treatment.

Case number	Duration of SLE (month)	Major current disease manifestations other than arthritis	Major previous disease manifestations other than arthritis	CTD other than RA or SLE	ANA	Anti-DNA antibody (U/mL)	Other autoantibodies	IgG (mg/dL)	C3 (mg/dL)	C4 (mg/dL)	CH50 (U/mL)	SLEDAI
1	2	Low grade fever, rash, anemia, leukocytopenia	Fever, rash, anemia, leukocytopenia	SS, chronic thyroiditis	>1280	29.5	Ro, LA, TPO	2285	81	14	36.8	7
2	565	Rash	Rash, leukocytopenia, pericarditis, pleuritis	SS	>1280	47.8	Ro, La	1265	87	12	31.1	12
3	20	None	Fever, anemia, leukocytopenia	None	320	28.3	U1-RNP, Cardiolipin	1939	27	3	<5.0	8
4	151	Rash	Rash, leukocytopenia	Scleroderma, chronic thyroiditis	>1280	16.8	Sm, U1-RNP, TPO	1625	111	41	47.5	6
5	420	None	Fever, rash, severe thrombocytopenia	SS	160	10.1	Ro, La	2285	66	4	17.4	6
6	96	None	Fever, rash, myositis, ILD	SS	320	4.0	U1-RNP, Ro	1598	152	40	45.1	4

SLE: systemic lupus erythematosus; CTD: connective tissue disease; ANA: antinuclear antibody; IgG: immunoglobulin G; SLEDAI: systemic lupus erythematosus disease activity index; ILD: interstitial lung disease; SS: Sjogren's syndrome; APS: antiphospholipid syndrome; LA: lupus anticoagulant; TPO: thyroid peroxidase; RNP: ribonucleoprotein.
